# Prevalence of oral mucosal lesions in children in Xiangyun of Yunnan, China: a cross-sectional study

**DOI:** 10.1186/s13052-022-01209-6

**Published:** 2022-01-29

**Authors:** Hui Yao, Qianqian Song, Qiongyue Zhang, Guoyao Tang, Mingshan Liu

**Affiliations:** 1 Department of Oral Medicine, College of Stomatology, National Center for Stomatology, Shanghai Ninth People’s Hospital, Shanghai Jiao Tong University School of Medicine, Shanghai Jiao Tong University, National Clinical Research Center for Oral Diseases, Shanghai Key Laboratory of Stomatology, Shanghai, China; 2 Department of Stomatology, the People’s Hospital of Xiangyun Affiliated To Dali University, Yunnan, China

**Keywords:** Oral mucosa, Child, Prevalence, Epidemiology, Rural

## Abstract

**Background:**

This population-based cross-sectional study aimed to evaluate the prevalence of oral mucosal lesions (OMLs) in children in a rural area in China as epidemiological data on these conditions from such areas are insufficient.

**Methods:**

A total of 3145 children in Xiangyun of Yunnan were enrolled. A socio-economic questionnaire and a field survey of OMLs were administered. We gathered information on factors (sex, age, caregiver, parental education, and last-month household income) that might be associated with OMLs in these children.

**Results:**

OMLs in children in Xiangyun of Yunnan had a prevalence of 1.8% (95% CI; 1.3–2.3%). The most prevalent OML was oral ulcer (*n* = 11; 18.3%), followed by linea alba (*n* = 10; 16.7%), whereas the least prevalent OMLs were frenal tag (*n* = 1; 1.7%) and herpes labialis (*n* = 1; 1.7%). On unadjusted or adjusted regression, school-aged children had about 50% lower risk of OMLs than preschoolers.

**Conclusions:**

These findings indicate that the prevalence of OMLs in Xiangyun of Yunnan is lower than previously reported. Additionally, the age might be associated with the occurrence of OMLs in children in Xiangyun of Yunnan, China.

**Supplementary Information:**

The online version contains supplementary material available at 10.1186/s13052-022-01209-6.

## Background

Oral mucosal lesions (OMLs) are a group of conditions that occur on the surface of the oral mucosa and present a variety of lesions. These lesions can impair the activities of chewing, swallowing, and speaking [1, 2]. The discomfort caused by OMLs requires extra attention, especially in children. Impairment and discomfort, along with possible psychological problems, might consequently distract children and lead to poor academic performance [3].

Internationally, studies have been conducted on the prevalence of OMLs to provide a practical strategy for their treatment and prevention. Most of these studies have focused on potentially malignant lesions, such as oral leukoplakia and oral lichen planus [4–6]. Less attention has been paid to nonneoplastic lesions, which constitute a considerable proportion of oral health problems worldwide [7]. In addition, related epidemiological data on OMLs in children from China are lacking, especially in remote and rural areas. Since socioeconomic status (SES) is a factor associated with the prevalence of OMLs [8], further understanding of this association is needed to inform the decisions of healthcare providers and policy makers.

In this study, we aimed to assess the prevalence of OMLs and explore the association between SES and oral mucosal health status in children in Xiangyun of Yunnan, China.

## Methods

### Participants

This population-based cross-sectional study consisted of a home questionnaire and a field survey conducted by the Department of Stomatology, People’s Hospital of Xiangyun affiliated with Dali University between September 2020 and January 2021. We calculated the minimum sample size using Epi Info 7 with a 95% confidence level, a 0.05 margin of error, a design effect of 2, a 69.5% reported prevalence of OMLs [8], and a 10% additional sample size to compensate for the possible sample loss. Finally, we obtained a minimum sample size of 724. We recruited approximately 4000 participants from four primary schools and six preschools located in four geographical regions (eastern, southern, western, and northern) in Xiangcheng Town, Xiangyun County in Yunnan Province through the 2020 National Oral Health Comprehensive Intervention Program for Children in China. The government of Yunnan Province announced that Xiangyun County had eliminated poverty in September 2018 [9]. The per capita disposable income of Xiangyun County in 2019 was 37,689 Yuan (4997 Euro) [10], equivalent to ~ 6000-Yuan per month per household from two full-time incomes. This study was approved by the Ethics Committee of the People’s Hospital of Xiangyun (No. 2020069). Informed consent was obtained from the guardians of all participants.

### Exposures

The home questionnaire (Additional file 1) was administered to the guardians, and consent was obtained at that time. Variables collected through the questionnaire consisted of participants’ basic information (sex, age group, and ethnicity), chronic disease history, and SES, which included whether children had siblings, caregivers, parental education, last-month household income, and residence status. The variables were considered as exposures to explore whether they were associated with OMLs morbidity.

### Outcomes

To define the OMLs, we applied the World Health Organization guidelines [11, 12] and referred to the textbook Oral and Maxillofacial Pathology [13]. Here, however, oral ulcer referred to any ulcer lesion in mouth due to a wide range of etiologies, including trauma, infection or inflammation.

In the presurvey preparation, a specialist provided a training program for five examiners by presenting 50 color images. These images covered the full spectrum of oral mucosal alterations and lesions expected during the survey. Following the training, the examiners continued learning until they obtained full marks during the testing session on the same images. Before starting a new-day survey, the examiners repeated the learning and testing processes. To achieve consistency, the inter-examiner Kappa value was calculated. Examinations assessing inter-agreement [14] took place in each examiner’s first five participants. The results were compared between examiners 1 and 2, 2 and 3, 3 and 4, 4 and 5, and 5 and 1. The Kappa scores were between 0.81 and 0.85, which met the agreement criteria.

During the survey, diagnoses were confirmed by a specialist when examiners were unsure of the findings. In the field survey, oral examinations were performed under artificial and natural light, with the children in a standing position. The examiners inspected and palpated using a mirror, explorer, cotton swab, and sterile gauze in the sitting position. The examination sequence was as follows: face, lip, buccal cavity, tongue, mouth floor, hard palate, soft palate, gum, and alveolar ridge. We adhered to the local hospital’s guidelines on infection control. At the end of our survey, we wrote down our suggestion of our findings on conclusion letters to guardians. The letters also stated the time and the place of our oral medicine service. Some children were treated by us, although some did not visit us.

### Statistical analysis

Categorical variables were expressed as numbers and percentages (%). Statistical significance was set at *p* < 0.05. We replaced missing data on caregivers (*n* = 132, 4.2%), parental education (*n* = 81, 2.6%), and las-month household income (*n* = 134, 4.3%) with the mode of each variable when doing unadjusted or adjusted regression to analyze the risk factors (sex, age, caregiver, parental education, and last-month household income) associated with the presence of OMLs among the children. All statistical analyses were performed using SPSS 26.0.

## Results

**Fig. 1:**
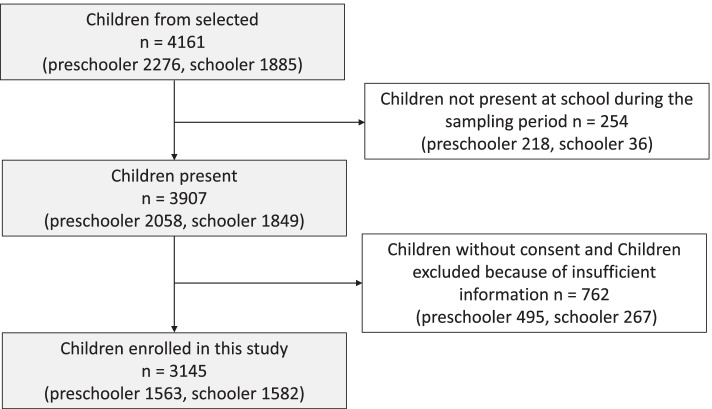
Flow chart of subjects’ enrollment

In total, 4161 children were invited to participate in our study. This population comprised 2276 preschoolers, aged 3–5 years and 1885 school-aged children, aged 7–8 years. However, only 3907 children (93.9%) were present in the study field (Fig. 1). Of the total of 4161 children, 762 (18.3%) including 495 preschoolers and 267 school-aged children were excluded. The exclusion criteria were lack of parental/guardian consent (*n* = 744) and insufficient data (*n* = 18) such as the absence of name, sex, or age in the questionnaire (Fig. 1).

The prevalence of OMLs in children in Xiangyun of Yunnan was 1.8% (95% CI, 1.3–2.3%), 1.2% (95% CI, 0.7–1.7%) in school-aged children and 2.4% (95% CI, 1.7%–3.2%) in preschoolers. The clinical characteristics of the study subjects are shown in Table 1. The ratio of boys to girls was 1634/1511, and the prevalence of OMLs was similar between boys (*n* = 29, 0.9%) and girls (*n* = 28, 0.9%). Half of the children were school-aged, and half were preschoolers; however, the number of preschoolers with OMLs was twice the number of school-aged children.

Of the 3145 children, only 600 (19.1%) were not of Han ethnicity, and 10 (0.3%) were of minor ethnicity with OMLs. Of the 2919 children, 802 (27.5%) belonged to an only-child family. Of the 3013 children, 2598 (86.2%) had parents as caregivers, with the highest level of education achieved as follows: 908 (29.6%) did not complete high school, 814 (26.6%) completed high school, and 1342 (43.8%) completed a college or university degree. The last month household income was as follows: 1701 (56.5%) earned less than 6000 Yuan, 810 (26.9%) earned 6000–12,000 Yuan, and 500 (16.6%) earned above 12,000 Yuan. Approximately one-third of the children were natives (*n* = 880; 32.9%). In contrast, half of the children with OMLs were natives and half of them were migrants (*n* = 29; 1.1%). In terms of chronic diseases, nine children without OMLs reported having leukemia (*n* = 1), Kawasaki disease (*n* = 1), cholelithiasis (*n* = 1), asthma (*n* = 2) and heart disease (*n* = 3).

Table 2 shows prevalence among 12 types of OMLs in Xiangyun of Yunnan in 2020. The most prevalent OML was oral ulcer (*n* = 11;18.3%), followed by linea alba (*n* = 10; 16.7%). The least prevalent OMLs were frenal tag (*n* = 1; 1.7%) and herpes labialis (*n* = 1, 1.7%), only about one tenth of the highest prevalence (Table 2).

**Table 1:** Variables of the child and oral mucosal lesions of the child

	Total	Non-OMLs	OMLs
**Sex**			
Boy	1634 (51.9)	1605 (51.0)	29 (0.9)
Girl	1511 (48.0)	1483 (47.2)	28 (0.9)
**Age**			
School	1582 (50.3)	1563 (49.7)	19 (0.6)
Preschool	1563 (49.7)	1525 (48.5)	38 (1.2)
**Ethnicity**			
Han	2545 (80.9)	2498 (79.4)	47 (1.5)
Others	600 (19.1)	590 (18.8)	10 (0.3)
**Only child**†			
Yes	802 (27.5)	786 (26.9)	16 (0.6)
No	2117 (72.5)	2076 (71.1)	41 (1.4)
**Caregiver**†			
Parents	2598 (86.2)	2551 (84.7)	47 (1.6)
Others	415 (13.8)	405 (13.4)	10 (0.3)
**Parental education**† **(highest level achieved)**			
Less than high school	908 (29.6)	895 (29.2)	13 (0.4)
High school diploma	814 (26.6)	801 (26.1)	13 (0.4)
College or university	1342 (43.8)	1311 (42.8)	31 (1.0)
**Last-month household income**†			
Less than 6000 Yuan	1701 (56.5)	1670 (55.5)	31 (1.0)
6000-12,000 Yuan	810 (26.9)	796 (26.4)	14 (0.5)
More than 12,000 Yuan	500 (16.6)	488 (16.2)	12 (0.4)
**Residence status**†			
Native	880 (32.9)	851 (31.8)	29 (1.1)
Migrant	1795 (67.1)	1766 (66.0)	29 (1.1)
Chronic disease*			
Yes	9 (0.3)	9 (0.3)	0 (0)
No	3136 (99.7)	3079 (97.9)	57 (1.8)

Data are n (% of available data). *OMLs* = oral mucosal lesions. The total number of participants was 3145. †Missing data: only child, *n* = 226, caregiver, *n* = 132, parental education, *n* = 81, last-month household income, *n* = 134, residence status, *n* = 470. *Chronic disease included leukemia (*n* = 1), Kawasaki disease (*n* = 1), cholelithiasis (*n* = 1), asthma (*n* = 2) and heart disease (*n* = 3)

**Table 2:** Distribution of oral mucosal lesions in the examined sample

OMLs	n	Relative frequency (%)	Overall percentage (%)	95%CI
Cheilitis	4	6.7	0.1	0–0.3
Coated tongue	4	6.7	0.1	0–0.3
Figured tongue	4	6.7	0.1	0–0.3
Frenal tag	1	1.7	0.0	0–0.1
Geographic tongue	5	8.3	0.2	0–0.3
Herpes labialis	1	1.7	0.0	0–0.1
Leukoedema	2	3.3	0.1	0–0.2
Linea alba	10	16.7	0.3	0.1–0.5
Oral ulcer	11	18.3	0.4	0.1–0.6
Pigmentation lesion	8	13.3	0.3	0.1–0.4
Tongue tie	8	13.3	0.3	0.1–0.4
Vascular lesion	2	3.3	0.1	0–0.2
Total	57	100	1.8	1.3–2.3

Data are n (% of available data). *OMLs* = oral mucosal lesions. The total number of OMLs was 60

On either unadjusted or adjusted regression, school-aged children had an approximately 50% lower risk of OMLs than preschoolers. In addition, there was no evidence of increased risk of OMLs with other factors, including sex, caregiver, parental education, and last-month household income (Table 3).

**Table 3:** Exposures associated with the presence of oral mucosal lesions in the examined sample

	Unadjusted OR (95%CI)	*p* value	Adjusted OR (95%CI)	*p* value
**Sex**				
Boy	0.96 (0.57–1.62)	0.87	0.95 (0.56–1.60)	0.84
Girl	Reference	-	Reference	-
**Age**				
School	0.49 (0.28–0.85)	0.01	0.52 (0.29–0.91)	0.02
Preschool	Reference	-	Reference	-
**Caregiver**				
Parents	0.71 (0.36–1.42)	0.33	0.79 (0.40–1.59)	0.51
Others	Reference	-	Reference	-
**Parental education**		0.37		0.60
Less than high school	0.65 (0.34–1.25)	0.20	0.72 (0.35–1.47)	0.37
High school diploma	0.73 (0.38–1.40)	0.34	0.77 (0.39–1.52)	0.45
College or university	Reference	-	Reference	-
**Last-month household income**		0.56		0.82
Less than 6000 Yuan	0.70 (0.36–1.37)	0.30	0.87 (0.42–1.79)	0.70
6000—12,000 Yuan	0.72 (0.33–1.56)	0.40	0.78 (0.36–1.70)	0.53
More than 12,000 Yuan	Reference	-	Reference	-

Adjusted for sex, age, caregiver, parental education and last-month household income

## Discussion

In this study, we found a lower prevalence (1.8%) of OMLs in Xiangyun of Yunnan than that in the previous study [15] and the most common OML was oral ulcer. Preschoolers had a greater risk of developing OMLs than did school-aged children. However, no other risk factors (including sex, caregiver, parental education, and last-month household income) for developing OMLs were found. These are major findings since OMLs in children in remote and rural areas are generally neglected and may impair oral health-related quality of life [16]. However, this can improve with proper prevention and early intervention.

There are a few studies on OMLs among children reporting prevalence ranging from 4.1% to 69.5%, despite some variations in different studies [8, 17]. In our current study, the most prevalent OML was oral ulcer, which was consistent with Kleinman’s study [17] and de Oliveira’s study [16], although the rate was relatively low (0.4%) in children aged 4–5 and 7–8 years. Kleinman et al. reported a 1.23% prevalence of oral aphthous ulcers in children aged 5–17 years [17]. A study from de Oliveira reported that ulcers were the most prevalent OMLs (29.4%) in children aged 5 years [16]. Nevertheless, recurrent aphthous stomatitis was the 2nd most prevalent OML (1.64%) in the Third National Health and Nutrition Examination Survey among 2–17 year-old children and youth [18]. According to Vieira-Andrade’s study [8], the most prevalent OMLs were coated tongue (23.4%), melanotic macules (14.4%), and oral ulcers (11.8%) in children aged 0–5 years. Of the children with oral ulcers, 65.6% belonged to the 3–5 year age group. Bessa et al. reported aphthous ulcerations as the 5th most prevalent OML in children aged 0–4 years (1.47%) and the 6th most prevalent in children aged 5–12 years (1.72%), whereas the most common lesion was the geographic tongue [19]. According to Hong’s study [15], oral mucocele was the most commonly oral mucosal lesion biopsied, although it was not the same case in 20 clinical studies. Our study also did not find any mucocele due to its cross-sectional nature. It is possible that diagnostic criteria, training of examiners, calibration of examination procedures, and enrolled participants could influence the results [20, 21]. The reason for the low occurrence of OMLs in this study might be partly because this region was covered by the National Oral Health Comprehensive Intervention Program for Children in China. Some aims of this program were to improve people’s knowledge, influence their attitudes, and modify their behavior toward oral health [22]. This might benefit children and reduce the prevalence of OMLs.

Previously, in terms of risk factors, a study also found that age between 3 and 5 years was the determining factor for OMLs [8] and another study from the US found that 5–11 year-old children had a significantly higher prevalence of OMLs than children aged 12–17 years [17]. Bessa et al. reported that older children (5–12 years) had a significantly higher occurrence of OMLs than younger children (0–4 years) group [19]. It is notable that children around 5 years of age are more vulnerable to OMLs. The reason could be milestone children reaching 5 years of age. Oral ulcers and herpetic infections are among the most common types of OMLs in children [8, 15]. Oral ulcers can be associated with psychological and mechanical stress [23]. For example, children around 5 years of age might struggle to adapt to new environments and be anxious or stressed about separation from their caregivers. As a result, anxiety or stress leads to the occurrence of oral ulcers. In addition, herpetic infections occur in children between 6 months and 5 years of age [13]. Due to its communicable nature, the incidence of herpetic infection could reach its peak in children aged around 5 years, which occurs during their preschool years.

It is the age that social factors shape health [24]. SES is associated with a variety of children’s health due to differences in access to medical services, social support, and reactions to stress [25]. Evidence shows that OMLs are associated with a low socioeconomic status [8]. For example, higher occurrence of coated tongue in children was associated with the low family income. However, we found this was not the case and our findings were consistent with those of a study by Bessa et al. [19]. An explanation could be that all children lived in the town, not in villages. The residents have all been successfully lifted out of poverty. As a result, they can obtain basic healthcare, social resources, and support to adjust to stress. In addition, the effects of SES on the occurrence of OMLs might not be too evident in children. All these factors might contribute to the lack of association between OMLs and SES.

One strength of our study on the prevalence of OMLs in children is that it was conducted in a rural area, as such studies are rare. Another strength is that we enrolled a large sample size. Our study, however, has some limitations due in part to the study design. Oral ulcers and herpetic infections are two recurrent types of OMLs that can heal without treatment. These were not present in children when we performed the examination. As a result, our study may have underestimated the number of children with these two lesions because of its cross-sectional nature. Furthermore, some information on SES was obtained from self-reported data. For example, some caregivers might have falsely reported lower last-month household income due to fear of losing welfare, such as the poverty allowance. It is possible that we had an increased number of households with an income of less than 6000 Yuan/month. Consequently, a regression analysis of household income could be unprecise.

With economic growth and social changes, children are vulnerable to OMLs [26]. Treatment and prevention of OMLs to make children free of pain, fear, and anxiety is the job of specialists in oral medicine. However, the number of specialists is inadequate to meet the demand for oral medicine care, especially in rural areas. Consequently, it is easy to miss good opportunities to diagnose and treat OMLs. In such circumstances, our data on OMLs among children in Xiangyun of Yunnan can help inform oral health prioritization necessary to prevent and treat oral diseases.

## Conclusions

This study demonstrated that OMLs have a low prevalence in children in Xiangyun of Yunnan. This suggests that there might be an association between age and the development of OMLs in children in Xiangyun of Yunnan, China.

## Supplementary Information

**Additional file 1.**

## Data Availability

The data and material gathered in this study can be requested from the corresponding author reasonably.

## Abbreviations

OMLs: Oral mucosal lesions
SES: Socioeconomic status
WHO: World Health Organization
