# Supplementary material for: Prevalence of oral mucosal lesions in children in Xiangyun of Yunnan, China: a cross-sectional study
Source: Ital J Pediatr. 2022 Jan 29;48:15. doi: 10.1186/s13052-022-01209-6 (PMC8800258; doi:10.1186/s13052-022-01209-6)
Supplement: Supplementary file 1 — Additional file 1. [file 13052_2022_1209_MOESM1_ESM.docx]

CHILD’S PERSONAL AND FAMILY HISTORY

1. SEX OF CHILD

□ Boy

□ Girl

2. CHILD’S AGE GROUP

□ School

□ Preschool

3. ETHNICITY

□ Han

□ Bai

□ Others

4. PERSON COMPLETING THIS FORM

□ Mother

□ Father

□ Grandparent

□ Others

5. THE ONLY CHILD IN THE FAMILY

□ Yes

□ No

6. PERSON BEING THE CAREGIVER

□ Mother

□ Father

□ Grandparent

□ Others

7. FATHER’S HIGHEST LEVEL OF EDUCATION

□ None

□ Lower than high school

□ High school or vocational training

□ Associate degree

□ Bachelor’s degree

□ Post-graduate

8. MOTHER’S HIGHEST LEVEL OF EDUCATION

□ None

□ Lower than high school

□ High school or vocational training

□ Associate degree

□ Bachelor’s degree

□ Post-graduate

9. LAST-MONTH HOUSEHOLD INCOME

□ < 3000 Yuan

□ 3000—6000 Yuan

□ 6000—12000 Yuan

□ 12000—20000 Yuan

□ ≥ 20000 Yuan

10. RESIDENCE STATUS

□ Migrant

□ Native

11. PAST MEDICAL HISTORY

□ Blood Disease

□ Asthma

□ Heart Problems

□ Epilepsy (Seizures)

□ Others
